# Of Snails, Earthworms, and Men: Insights into Strategies to Preserve Water

**DOI:** 10.1093/function/zqab071

**Published:** 2021-12-23

**Authors:** Christian Delles, Giacomo Rossitto

**Affiliations:** Institute of Cardiovascular and Medical Sciences, University of Glasgow, Glasgow, Scotland, UK; Institute of Cardiovascular and Medical Sciences, University of Glasgow, Glasgow, Scotland, UK; Department of Medicine, University of Padua, Padua, Italy

**A Perspective on “The contribution of plasma urea to total osmolality during iatrogenic fluid reduction in critically ill patients”**

The evolution from life in oceans where water is abundant to life on land where water is rare required the development of water-preserving mechanisms. What is true for water is also true for sodium: It is highly abundant in the sea and rare at land. Terrestrial life can, therefore, focus on preservation of sodium and osmotically, water will follow. This is admittedly an oversimplistic description of sodium and fluid homeostasis but it remains one of the key tasks of the kidneys that spend majority of energy on tubular reabsorption of sodium.^1^

Terrestrial animals developed a number of strategies to preserve water. The basic principles of renal function are remarkably conserved across all forms of life indicating the importance of salt and water homeostasis for all animals. However, animals that live in particularly dry and/or hot areas developed further strategies including modifications to the renal tubular system to further optimise sodium reabsorption, excretion of nitrogen in the form of uric acid rather than urea, and of course physical barriers including the makeup of the skin to reduce transdermal fluid loss and perspiration.

The basic principle that for osmotic reasons, salt and water go hand-in-hand has been key to our understanding of cardiovascular and renal physiology.^2^ This principle has, however, been challenged. For example, whilst urinary sodium excretion generally mirrors sodium intake, sodium excretion follows a rhythmicity and cyclical pattern that is, on a daily basis, not fully in line with sodium intake.^3^ The concept of non-osmotic sodium storage particularly in the skin has further challenged the tight links between salt and water.^4^ Sodium also plays important roles beyond being an osmolyte and a carrier of a positive charge, for example by interacting with the immune system.^5^ Whilst these findings indicate that the link between water and salt could be less tight than originally thought, they also open the possibility that other osmolytes play a prominent role in “binding” free water.

In its extreme form, this role is evident in aestivating organisms. Aestivation is a dormant state not unlike hibernation with reduced metabolism and activation of defence mechanisms against oxidative stress to preserve organs for prolonged periods of time. One of the key challenges is preservation of water, and metabolic changes to increase levels of urea and glucose to act as non-ionic osmolytes are, therefore, seen in aestivating animals.^6^ Whilst aestivation is typically seen in invertebrates, it has also been described in vertebrates and even some mammals.

Humans are no exception and face similar challenges. They live in almost every climate zone and their kidneys are extremely efficient in preserving sodium and water. Even in demanding physiological situation such as pregnancy with its increased cardiac output and expansion of total body volume, adaptations of the kidneys, and the renin–angiotensin–aldosterone system (RAAS) help to maintain fluid homeostasis and intravascular volume.^7^ There are, however, pathological conditions that are so extreme that physiological response mechanisms fail. Patients with these conditions are typically admitted to high dependency or intensive care units.

One of the most important goals of intensive care medicine is to maintain blood pressure, and thereby perfusion pressure of critically important organs. Blood pressure is the product of cardiac output (or more generally: Intravascular volume) and vascular resistance, and both factors can be pathologically altered in critically ill patients. Conditions such as haemorrhage or diarrhoea lead to absolute loss of volume, vasodilation and vascular leakage in sepsis leads to both reduced vascular resistance and reduced intravascular volume, and cardiac failure leads to reduced cardiac output. There are also conditions that increase total body fluid but not necessarily intravascular volume, namely kidney or liver failure. Administration of large amounts of intravenous fluids, generally in the form of normal saline, is a common therapeutic strategy to increase intravascular volume, and thereby maintain cardiac output and blood pressure.

This initial fluid resuscitation comes with a price. Once haemodynamically stable, patients will often face several litres of volume overload that cannot be offloaded quickly due to changes in renal function and activation of water preserving mechanisms such as the RAAS. Therefore, an offloading strategy, typically facilitated by diuretics or haemofiltration, often forms part of patient management in the following days.

In this issue of *Function*, Sandra Nihlén and colleagues^8^ hypothesised that patients who experience fluid losses also activate systems akin to those observed in aestivating animals. Their paper is elegantly written and needs no detailed explanation here. Just a reminder: In aestivation we would expect an increase in total osmolality, driven by an increased contribution of non-ionic (organic) osmolytes, and a relative or absolute reduction in ionic (particularly sodium) osmolytes. Nihlén *et al*.^8^ studied 241 critically ill patients in the postresuscitative phase who had an initial event that required administration of large amounts of fluids and were now in a period of fluid offloading. Of course, they found that total osmolality increased in this phase, and this will be largely driven by a net loss of water. However, the authors convincingly showed that the changes in osmolality are associated with reduced contributions of sodium and increased contributions of urea to osmolality, exactly what one would see in aestivation. It is worth reading their paper in detail as the degree of upregulation of this system in order to increase osmolality was found to be a determinant of survival in these patients, a finding that goes beyond the scope of this commentary but highlights the importance of endogenous water preserving mechanisms as possible clinical outcome predictors.

Do the results of the study by Nihlén *et al*.^8^ come with any practical consequences? Maybe. It is worth drawing the reader's attention to two of the clinical perspectives in the paper. First, the increased urea levels are the consequence of catabolism, and hence muscle wasting. Muscle wasting is a common finding in hospitalised patients and particularly in the intensive care setting. Nihlén *et al*.^8^ highlight a potentially important mechanism for protein catabolism in patients experiencing active or passive fluid losses. Further to this, the authors convincingly show that the increase in serum urea concentration is not driven by external nitrogen load (ie nutrition), but by catabolism driven by the critical illness. To add further complexity to the picture, we and others have recently shown that sodium load is associated with protein catabolism,^9,10^ indicating that urea and sodium are connected in many ways in acute and chronic disease. What exactly this means to nutrition strategies remains unclear at this stage. What is clear, however, is that patients who just experienced a major challenge to fluid homeostasis due to critical disease should, where possible, not undergo yet another event of aggressive dehydration in the postresuscitative phase of their disease.

The paper by Nihlén *et al*.^8^ comes with many limitations. These range from the retrospective and somewhat observational nature of the study to limitations with precise measurements of key parameters including some of the osmolytes and osmolality itself. Despite the limitations, however, the data are clear and convincing. So, rather than dwelling of apparent limitations, we would like to congratulate the authors on a piece of physiology that they delivered by carefully studying patients in an extreme condition.

Research in the intensive care setting is challenging. Clearly, patient care is of utmost importance and in a highly demanding clinical setting there is limited time for deliberation and complex clinical studies. The heterogeneity of patients in this setting with multiple acute and chronic pathologies is often seen as a limitation and discourages some intensivists from mechanistic research. Nihlén *et al*.^8^ have impressively demonstrated that research is possible in this setting and that it can take advantage of the extremes of physiology that patients experience during critical illness. The recipe is simple: Focus on mechanisms that could play a role across multiple pathologies; define a clear-cut research question based on understanding of physiology and pathophysiology; and include a sufficiently large number of patients in order to compensate for some of the inherent limitations of such research.

The present paper by Nihlén *et al*.^8^ followed this recipe. We learned that humans share key physiological mechanisms with aestivating animals such as snails, earthworms, and frogs. The paper reminds us of common principles of life.

## Funding

We are grateful to the British Heart Foundation for supporting our work (Centre of Research Excellence Award RE/18/6/34217).

## References

[1] Deetjen P , Kramer K. Die Abhängigkeit des O_2_-Verbrauchs der Niere von der Na-Rückresorption. Pflugers Arch Gesamte Physiol Menschen Tiere. 1961;273(6):636–650. 13884819

[2] Rossitto G , Mary S, Chen JY et al. Tissue sodium excess is not hypertonic and reflects extracellular volume expansion. Nat Commun. 2020;11(1):4222. 32839436 10.1038/s41467-020-17820-2 PMC7445299

[3] Lerchl K , Rakova N, Dahlmann A et al. Agreement between 24-hour salt ingestion and sodium excretion in a controlled environment. Hypertension. 2015;66(4):850–857. 26259596 10.1161/HYPERTENSIONAHA.115.05851 PMC4567387

[4] Titze J , Lang R, Ilies C et al. Osmotically inactive skin Na+ storage in rats. Am J Physiol Renal Physiol. 2003;285(6):F1108–F1117. 12888617 10.1152/ajprenal.00200.2003

[5] Wilck N , Balogh A, Markó L et al. The role of sodium in modulating immune cell function. Nat Rev Nephrol. 2019;15:(9):546–558. 31239546 10.1038/s41581-019-0167-y

[6] Storey KB , Storey JM. Aestivation: signaling and hypometabolism. J Exp Biol. 2012;215(9):1425–1433. 22496277 10.1242/jeb.054403

[7] Gennari-Moser C , Escher G, Kramer S et al. Normotensive blood pressure in pregnancy: the role of salt and aldosterone. Hypertension. 2014;63(2):362–368. 24296282 10.1161/HYPERTENSIONAHA.113.02320

[8] Nihlén S , Frithiof R, Titze J et al. The contribution of plasma urea to total osmolality during iatrogenic fluid reduction in critically ill patients. Function. 2021;3:(1):zqab055. 35330925 10.1093/function/zqab055 PMC8788870

[9] Kitada K , Daub S, Zhang Y et al. High salt intake reprioritizes osmolyte and energy metabolism for body fluid conservation. J Clin Invest. 2017;127(5):1944–1959. 28414295 10.1172/JCI88532 PMC5409074

[10] Rossitto G , Maiolino G, Lerco S et al. High sodium intake, glomerular hyperfiltration, and protein catabolism in patients with essential hypertension. Cardiovasc Res. 2021;117(5):1372–1381. 33053160 10.1093/cvr/cvaa205 PMC8064429

